# Control of porcine reproductive and respiratory syndrome (PRRS) through genetic improvements in disease resistance and tolerance

**DOI:** 10.3389/fgene.2012.00260

**Published:** 2012-12-14

**Authors:** Raymond R. R. Rowland, Joan Lunney, Jack Dekkers

**Affiliations:** ^1^Department of Diagnostic Medicine and Pathobiology, College of Veterinary Medicine, Kansas State UniversityManhattan, KS, USA; ^2^Animal Parasitic Diseases Laboratory, United State Department of Agriculture, Agricultural Research Services, Beltsville Agricultural Research CenterBeltsville, MD, USA; ^3^Department of Animal Science, Iowa State UniversityAmes, IA, USA

**Keywords:** porcine reproductive and respiratory syndrome, PRRS resistance, genome-wide association study

## Abstract

Infections caused by porcine reproductive and respiratory syndrome virus (PRRSV) have a severe economic impact on pig production in North America, Europe, and Asia. The emergence and eventual predominance of PRRS in the 1990s are the likely result of changes in the pork industry initiated in the late 1970s, which allowed the virus to occupy a unique niche within a modern commercial production system. PRRSV infection is responsible for severe clinical disease, but can maintain a life-long subclinical infection, as well as participate in several polymicrobial syndromes. Current vaccines lessen clinical signs, but are of limited use for disease control and elimination. The relatively poor protective immunity following vaccination is a function of the virus's capacity to generate a large degree of genetic diversity, combined with several strategies to evade innate and adaptive immune responses. In 2007, the PRRS Host Genetics consortium (PHGC) was established to explore the role of host genetics as an avenue for PRRS control. The PHGC model for PRRS incorporates the experimental infection of large numbers of growing pigs and has created the opportunity to study experimental PRRSV infection at the population level. The results show that pigs can be placed into distinct phenotypic groups, including pigs that show resistance (i.e., low virus load) or pigs that exhibit “tolerance” to infection. Tolerance was illustrated by pigs that gain weight normally in the face of a relatively high virus load. Genome-wide association analysis has identified a region on chromosome 4 (SSC4) correlated with resistance; i.e., lower cumulative virus load within the first 42 days of infection. The genomic region is near a family of genes involved in innate immunity. The region is also associated with higher weight gain in challenged pigs, suggesting that pigs with the resistance alleles don't seem to simultaneously experience reduction in growth, i.e., that resistance and tolerance are not antagonistically related. These results create the opportunity to develop breeding programs that will produce pigs with increased resistance to PRRS and simultaneously high growth rate. The identification of genomic markers involved in actual tolerance will likely prove more difficult, primarily because tolerance is difficult to quantify and because tolerance mechanism are still poorly understood. Another avenue of study includes the identification of genomic markers related to improved response following vaccination.

## Introduction

Porcine reproductive and respiratory syndrome (PRRS) is the most economically significant disease impacting commercial pig production in North America, Europe, and Asia. PRRS was first described in 1987, followed by characterization of the PRRS virus (PRRSV) in Europe in 1991, and soon after in the US (Benfield et al., 1992). Clinical outcomes following infection include reproductive failure, respiratory disease in young pigs, and reduced growth performance (Keffaber, 1989; Zimmerman et al., 2006; Lunney et al., 2010). Perhaps the greatest impact is the maintenance of a relatively long-term subclinical infection which participates in a variety of polymicrobial syndromes, such as porcine respiratory disease complex (PRDC) and porcine circovirus associated disease (PCVAD). In the field, PRRSV continues to be linked to a variety of new disease syndromes. In 2005, the emergence of porcine high fever disease (PHFD) in China was linked to a novel PRRSV strain (Tong et al., 2007). Affected herds experienced high morbidity and in some cases 100% mortality. PHFD PRRSV continues to spread throughout Southeast Asia and has been linked to the co-infection of pigs with the *Reston ebolavirus* (Barrette et al., 2009; Rowland et al., 2012). A new PRRS virus, called Lena, is associated with outbreaks of severe PRRS in Europe (Karniychuk et al., 2010).

PRRSV is a member of the arterivirus group, which includes lactate dehydrogenase–elevating virus (LDV) of mice, simian hemorrhagic fever virus (SHFV), and equine arteritis virus (EAV). The arteriviruses belong to the family, *Arteriviridae*, within the order, *Nidoviridales*. As a group, the arteriviruses possess several novel properties related to viral pathogenesis, including cytopathic replication in macrophages, the capacity to establish a persistent infection, and cause severe disease (Snijder and Spaan, 2007). The 15.4 kb PRRSV genome codes for at least 10 open reading frames (ORFs). The virion includes a nucleocapsid composed of a single nucleocapsid (N) protein. The viral envelope is dominated by the major glycoprotein, GP5, and the matrix (M) protein. Minor outer proteins include GP2, GP3, and GP4, along with two small proteins, E and ORF5 (Johnson et al., 2011). PRRSV is divided into European and North American genotypes, designated as type 1 and type 2, respectively. Even though type 1 and type 2 viruses were recognized almost simultaneously on the continents of North America and Europe and produce similar clinical signs, the two genotypes possess only about 70% identity at the nucleotide level. Nucleotide sequence diversity within each genotypic group can be as much as 10%. During the mid-1990s, viruses of type 2 origin were introduced into Europe. In 1999, type 1 viruses first appeared in North America (Fang et al., 2007).

The various clinical outcomes following PRRSV infection are a consequence of a complex set of interactions between the virus and the pig host. Following infection, viremia in young pigs continues for ~28 days. During this time, the virus primarily targets macrophages in the lung. The inflammatory response resulting from the infection and removal of alveolar macrophages is responsible for the onset of acute respiratory signs. Following the disappearance of virus from the blood, virus replication continues within monocyte/macrophage cells in the lymphoid tissues, including tonsils and lymph nodes (Rowland et al., 2003). Virus can be isolated from lymph nodes for more than 100 days after infection and persistently infect pigs is easily transmit virus to naïve pigs, likely via shedding from tonsils. Virus replication in the host gradually decays until the virus becomes extinct, at around 200 days after infection (Horter et al., 2001; Rowland et al., 2003). The mechanism for virus extinction is not clear, but likely relates to the gradual disappearance of PRRSV-permissive cells combined with a partially effective immune response; e.g., low levels of circulating neutralizing antibody. By definition, PRRSV is not a “persistent” virus, but since the typical lifespan of a commercial production pig is approximately 180 days, PRRSV infection is considered to be “life-long.”

The mechanistic basis for maintaining a life-long infection is dependent on a variety of factors, including; (1) a complex virion structure dominated by heavily glycosylated surface proteins, (2) the re-direction of the humoral response toward non-surface proteins, such as N and a variety of non-structural proteins, (3) antigenic and genetic drift within structural and non-structural genes, and (4) subversion of innate responses (Chand et al., 2012). Modified-live virus (MLV) and inactivated virus are the two principal approaches for PRRS vaccination. At least 20 PRRS vaccines are commercially available, worldwide. In general, inactivated virus vaccines are not effective. MLV vaccines are effective in protecting the pig from challenge with a genetically similar or “homologous” virus, but provide little protection against heterologous (genetically diverse) PRRSV isolates (Huang and Meng, 2010; Murtaugh and Genzow, 2011). The purpose of this review is to provide an overview of experimental models of PRRS infection, including the phenotypic properties of resistant and tolerant pigs. For the purpose of this review, PRRS “resistance” is defined as the ability of a host to limit pathogen burden, e.g., by inhibiting pathogen entry or restricting reproduction of the pathogen within the host and includes all mechanisms that limit the host pathogen burden. “Tolerance” is defined as the ability of a host to limit the detrimental impact of a given pathogen burden on the host's performance without directly affecting pathogen burden. Furthermore, for the purpose of this review, the definition includes the ability to maintain homeostasis in the presence of a replicating pathogen, with limited ensuing pathology.

## The role of the host genome in response to PRRSV infection

Since the discovery of the PRRS virus, there has emerged a body of evidence associating host genetics with different outcomes following PRRSV infection. In 1998, Halbur et al. evaluated PRRSV infection in a variety of pig breeds and reported more PRRS-associated lung lesions in Hampshire pigs. On the reproductive side, Lowe et al. (2005) concluded that genetics influenced abortion rates in PRRSV-infected Sows. Using an *in vitro* approach, Vincent et al. (2006) reported that macrophage responses were partially predictive of breeds with increased PRRSV resistance. Petry et al. (2005) found that, compared to a Hampshire/Duroc line, a Large White/Landrace line showed reduced viremia when infected with PRRSV. In later work, the same group (Petry et al., 2007) found that pigs with lower viremia possessed higher levels of serum interleukin-8 prior to infection. Previous estimates of heritability of PRRSV resistance are scarce, but heritability estimates for the effect of PRRSV infection on the percentage of live pigs born to infected sows range from 0.12 to 0.15 (Lewis et al., 2009). A recent review by Lunney and Chen (2010) describes the latest progress on the genetics of disease resistance, including the application of new tools such as the 60K SNP chip for performing genome-wide association studies (GWASs).

Studies related to understanding the genetic basis for PRRSV tolerance are non-existent. However, previous studies have shown that the detrimental impact of PRRSV infection on growth varies between and within lines and breeds (Greiner et al., 2000; Petry et al., 2005; Doeschl-Wilson et al., 2009), which may be considered as indication that genetic variation in tolerance exists.

A good example of tolerance is found in another arterivirus, LDV. Within 24 h after infection of a mouse, LDV infection levels in the blood approach 10^10^ virus particles per ml; however, there are no clinical signs of infection. Viremia decreases to about 10^7^ virus particles and remains at that level for the remainder of the mouse's life (Plagemann et al., 1995). The only evidence of infection is increased circulating lactate dehydrogenase (LDH), the result of a targeted elimination of a subpopulation of LDH-scavenging macrophages by LDV. The virus does not target the macrophage precursor; therefore, the level of LDV is maintained at a steady state depending on the production of new LDV-permissive macrophages. Normally, macrophages would be protected by the presence of virus-specific neutralizing antibody. Similar to PRRSV, the LDV-specific neutralizing antibody response is relatively weak, a consequence of a complex virion structure, including large quantities of surface protein glycosylation. Since the mouse does not become immunocompetent until after birth, mice can be made immunologically tolerant to LDV, by infecting neonates within 24 h after birth. The outcome of neonatal infection is the absence of a LDV-specific antibody response, a demonstration of immunological tolerance. However, in mice made immunologically tolerant to LDV, there is no alteration in the level of virus in the blood and no change in the course of viremia. Furthermore, the immunologically tolerant mice do not exhibit clinical disease signs (Rowland et al., 1994). Therefore, mice are “tolerant” to LDV infection. Tolerance to LDV infection is a mechanism that has the least impact on evolutionary fitness of the host. In a similar manner, a PRRSV tolerant pig would likely possess a relatively high virus load, but would show no pathology or clinical signs related to disease, including little reduction in growth or reproductive traits. In the real world, a PRRS tolerant pig would be particularly beneficial in high-density pig growing regions where PRRS is endemic and difficult to control. However, one unintended consequence of a pig showing tolerance would be the continuous shedding of virus, an efficient mechanism for spreading virus to naïve pigs.

In 2007, it was generally recognized by commodity groups, industry, and scientific communities that the next generation of improved PRRS vaccines was still years away and that genetic improvement offered a logical solution. In response, the National Pork Board supported the formation of the PRRS Host Genetics Consortium (PHGC). The PHGC was formed as a mechanism for conducting the scientific research necessary to elucidate the role of the host genome in the response of pigs to PRRSV infection. The ultimate goal is to find genomic markers that can be employed in the development of breeding programs to lessen the impact of PRRSV on the commercial pig industry. Genetic improvement does not offer a single “magic bullet” solution, but would be an integral component of other disease management strategies. For example, identification of genomic markers associated with enhanced protection after vaccination could be used to select for, and, so-called “vaccine-ready” pigs. As discussed above, pigs tolerant to PRRSV infection would offer a solution for regions with high pig densities where disease control is difficult. And finally, markers associated with susceptibility to disease would be useful to avoid the unintended consequences that can occur when breeding pigs for other desirable traits.

## Experimental models for investigating the role of the host genome in responses to PRRSV infection

For livestock species, investigating the association between genomic markers and the host response to infection typically incorporates hundreds, if not thousands, of infected animals. In the field, these numbers are readily achieved on affected farms by collecting phenotypic data, such as virus infection status (infected versus not infected animals), morbidity/mortality, and the presence or absence of clinical signs. Even though field data are highly relevant, assessing phenotypic traits associated with PRRSV infection can be complicated by several factors. For instance, obtaining only a single measure of infection status cannot be used to establish when the pig was first exposed or whether the infection is acute or chronic. Furthermore, the presence of other pathogens circulating within the population can mimic or mask PRRS clinical signs. For example, infection by influenza virus can mimic PRRSV respiratory clinical signs. Other complicating factors include the unknown properties of a particular PRRSV field isolate, the contribution of the environmental factors and overall health status.

The use of experimental infection models can eliminate or minimize the shortcomings of field studies. For instance, performing repeated phenotypic measurements following experimental challenge with a defined virus can yield reproducible and accurate determinations of virus-related traits, such as virus load, peak viremia, and viral clearance from the blood. Disease-related impacts on growth and performance can also be accurately measured. Other factors, such as nutrition and environment can be easily controlled. However, there are important considerations when performing experimental studies. For example, achieving the desired number of animals can be expensive. Another consideration is that experimentally infected animals maintained under “pristine” environmental conditions do not reproduce the environment found on the typical farm. Therefore, a particular experimental model may not reflect the response of animals maintained in the field.

Models that reproduce the effect of PRRSV on pregnant sows have been described in the literature (Rowland et al., 2003; Rowland, 2010). Measurable outcomes include the number of abortions or dead pigs. However, conducting pregnant sow studies on a large scale can be complex and prohibitively expensive. Another experimental PRRS model, often described in the literature measures the impact of PRRSV infection on the severity of respiratory disease in young pigs. Phenotypic disease traits include measurements of lung lesions scores and the amount of virus in alveolar macrophages, obtained by lung lavage. Pigs are experimentally infected and lungs removed between 7 and 15 days after infection. Phenotypic disease traits, such as lung lesions scores are obtained post-mortem. One limitation to this approach is the subjective nature for assessing lung pathology, which requires a pathologist or other trained professional to make the lung lesion determinations. Furthermore, different observers can obtain different disease scores for the same animal. The terminal nature of the experimental model prevents the collection of repeated measurements of lung lesion development and the resolution in the same pig.

The model developed by the PHGC incorporates a nursery pig model, described in Boddicker et al. (2012). High-health pigs are obtained from crossbred commercial lines with complete parentage and pedigree records. Pigs are negative for PRRSV, *Mycoplasma hyopneumoniae*, swine influenza virus (SIV), and porcine circovirus type 2 (PCV2). Each challenge trial group is comprised of a population of 200 pigs from at least 30 litters (six pigs per litter), which are derived from a minimum of 10 sires mated with 3–8 dams/sire. There is no pre-selection of sires or dams for any PRRS-related trait. Piglets and parents are genotyped for >60,000 single-nucleotide polymorphisms (SNPs) using the Porcine SNP60 BeadChip (Illumina). All phenotypic and genotypic data are stored and made available to the PHGC membership through a secure relational database (http://www.animalgenome.org/lunney/index.php).

Pigs, at 3–4 weeks of age, are challenged with a well-characterized PRRSV isolate. Infection and disease-related phenotypic traits are collected for 42 days after infection. The 42 day period covers both the acute and early persistent stages of PRRSV infection. For the purpose of definition, virus recovered from tonsil or other lymphoid tissues at 42 days of infection is the result of “persistence.” Virus load and weight gain are the two principal quantitative phenotypic traits measured, each reflecting important aspects of PRRSV infection. Virus load relates to amount of virus replication and reflects the potential for a pig to spread virus. Virus load is measured as the area under the curve of viremia measurements taken over the first 21 days after infection. Weight gain is used as measurement of the impact of that PRRSV infection has on growth performance. Both traits are quantifiable, easily reproducible, and do not require a high level of expertise to measure. Additional phenotypic data include measurements of innate and adaptive immunity, mortality, and the amount of virus in tonsil at 42 days.

## Phenotypic responses to PRRSV infection

**Figure 1:**
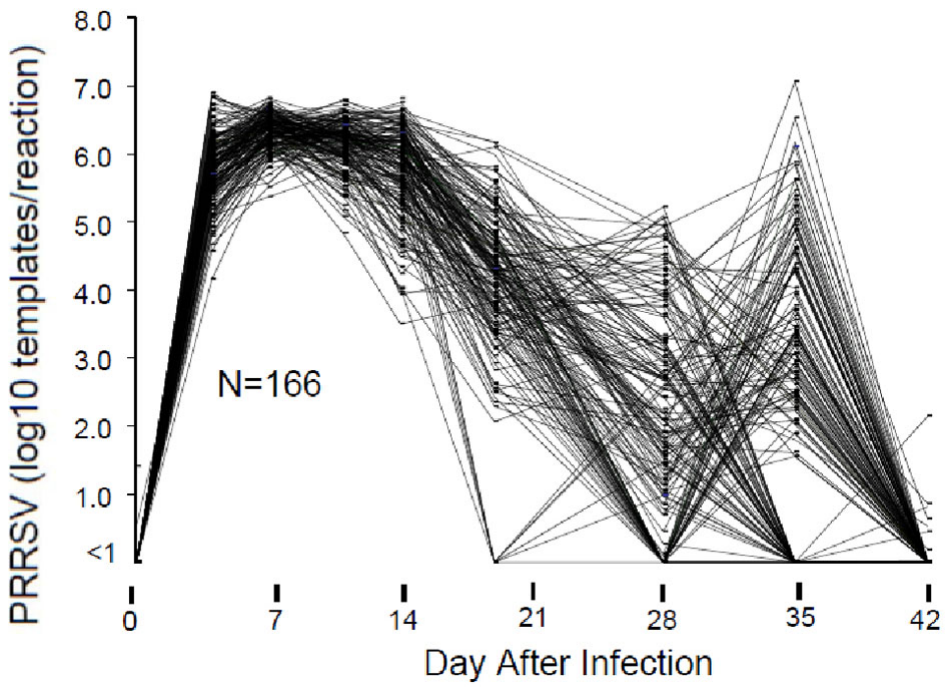
**Viremia following PRRSV infection.** Viremia was measured by RT-PCR of viral RNA using a commercial diagnostic PRRSV assay. For the purpose of standardization, the results were reported as number of PRRSV templates per 50 ul PCR reaction. Results are shown for those pigs in a single 200 pig trial that possessed data for all days.

The course of viremia and weight gain after experimental PRRSV infection are described by Boddicker et al. (2012). Figure 1 shows an example of PRRSV RT-PCR results for 166 pigs in a single experimental infection trial. In this example, viremia peaked between 7 and 14 days after infection and then declined. As expected, by 28 days post-infection, serum virus declined to undetectable levels in most pigs. However, in a subpopulation consisting of ~10–20% of pigs, circulating virus reappeared. Virus rebound following PRRSV infection is a phenomenon previously reported by Reiner et al. (2010). The mechanism for virus rebound is unclear, but could represent the emergence of immune escape variants.

**Figure 2:**
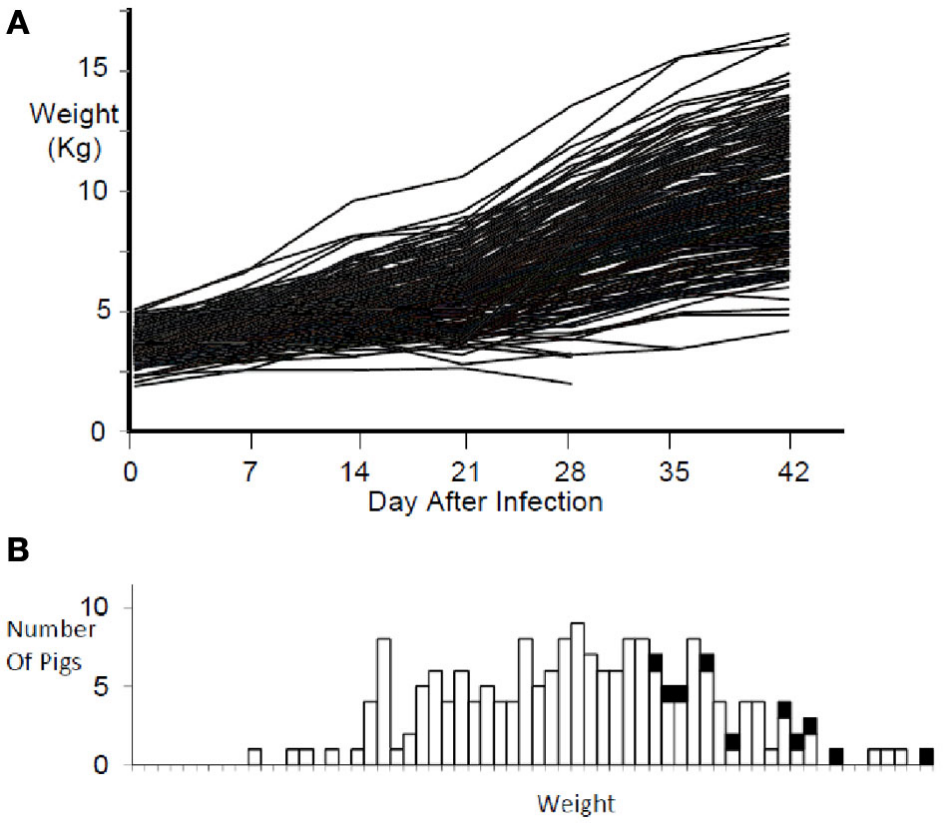
**Weight gain during PRRSV infection.** Panel **(A)** shows the weight gain for individual infected pigs for 42 days. Panel **(B)** shows the weight distribution at 42 days after infection for the same pigs in panel **(A)**. Black squares represent non-infected reference pigs.

**Figure 3:**
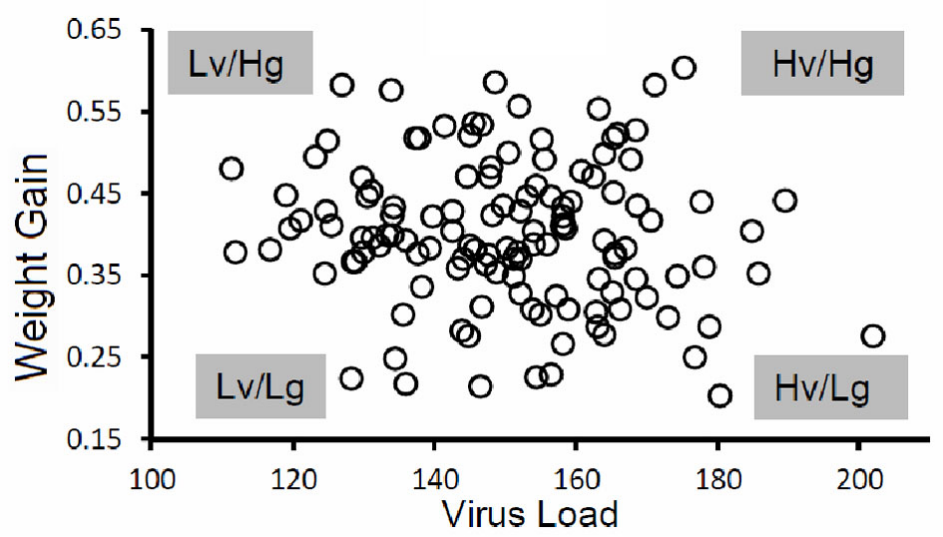
**Virus load versus weight gain.** The figure shows results for a single trial. The virus load was calculated as the area under the curve for viremia over the first 21 days for each pig as described in Figure 1. Average daily gain was calculated as the weight at 42 days after infection minus the weight on the day of virus challenge divided by 42 days. Key: Hv, high virus load; Lv, low virus load; Hg, high weight gain; Lg, low weight gain. Virus load for each pig was determined by calculating the area under the curve for the first 21 days after infection.

Pigs in the PHGC model showed a wide variation in weight gain, with some pigs gaining weight at a relatively normal rate, while others failed to thrive during the 42 day infection period (Figure 2). One hypothesis is that pigs that gain weight normally are the best at controlling virus infection. To address this possibility, a plot showing weight gain versus virus load is presented in Figure 3. The pigs falling at each of the four extremes of the scatter plot can be described as: high virus/low weight gain (Hv/Lg), high virus/high weight gain (Hv/Hg), Low virus/Low weight gain (Lv/Lg), Low virus/High gain (Lv/Hg). Approximately 10% of pigs fall into each of the extreme groups. Pigs in the Lv/Hg group can be described as “resistant” to the effects of PRRSV; whereas, Hv/Lg pigs are sensitive to infection. Pigs in the Hv/Hg qroup provide the best evidence for a subgroup of pigs that may be considered as “PRRSV tolerant,” i.e., retain normal growth in the presence of a relatively high virus load. However, caution is advised in the interpretation of the results as high growth rates alone are not necessarily reliable indicators of tolerance. In order to obtain unbiased tolerance estimates growth rate measures of the same pigs both infected and in the absence of the infection would need to be integrated into the appropriate statistical framework (e.g., Kause, 2011; Doeschl-Wilson et al., 2012).

## Genomic markers related to weight gain and virus load

To date, 11 groups of 200 pigs from seven genetic sources have been evaluated under the PHGC model. Results for the genetic analysis of the first 3 groups (PHGC1-3) from a single genetic source, are reported in Boddicker et al. (2012). The estimated heritabilities are 0.3 for both viral load and weight gain after challenge with the PRRSV isolate NVSL 97-7985 (Boddicker et al., 2012). A GWAS incorporating the 60 K SNP chip identified genomic regions associated with viral load on chromosomes 4 (SSC4) and SSCX regions on chromosomes SSC1, SSC4, SSC7, and SSC17 were associated with weight gain. Furthermore, both virus load and weight gain were associated with a single genomic region in SSC4, which is best represented by a single SNP marker, WUR10000125 (WUR). The 1 Mb region in SSC4, which exhibits strong linkage disequilibrium, explained 15.7% of the genetic variance for viral load and 11.2% for weight gain. The estimated effects for this region were favorably and nearly perfectly correlated; i.e., pigs with low virus load exhibited greater weight gain. The favorable allele (B) had a frequency of 0.16 within the experimental population of pigs. Although the number of individuals with the BB genotype was present at a low frequency, the B allele appeared to be dominant, i.e., pigs with the AB genotype showed a favorable response compared to AA.

Candidate genes near the WUR SNP include the guanylate-binding protein (GBP) gene family [reviewed in Vestal and Jeyaratnam (2011)]. GBPs are induced by cytokines, such as interferon, and are unique in their ability to bind guanylate. In mice, the family consists of 11 genes. Expression of GBP is associated with defense against a variety of RNA viruses, including hepatitis C virus, vesicular stomatitis virus, and encephalomyocarditis virus. The mechanism of how GBP might inhibit PRRSV replication or influence growth are unclear. The marker on SSCX, which was associated with only virus load, is in the region of CHST7, another gene with antiviral properties (Nyberg et al., 2004).

The results reported by Boddicker et al. (2012) provide the first clear evidence for a genomic marker linked to the response of the host to PRRSV infection and create the possibility to breed pigs for increased resistance to infection and improved performance. Unexpectedly, both disease traits converged at a single marker. In the experimental studies, genotype BB and AB pigs exhibited as much as 10% greater weight gain compared to the predominant AA genotype. This benefit is highly significant in an industry that survives on small profit margins. Future work is directed at determining if similar differences between AA and BB animals occur under field conditions as well as the investigation of other markers associated with disease resistance or tolerance.

### Conflict of interest statement

The authors declare that the research was conducted in the absence of any commercial or financial relationships that could be construed as a potential conflict of interest.
